# Participation trends in holistic movement practices: a 10-year comparison of yoga/Pilates and t’ai chi/qigong use among a national sample of 195,926 Australians

**DOI:** 10.1186/s12906-017-1800-6

**Published:** 2017-06-06

**Authors:** Ineke Vergeer, Jason A. Bennie, Melanie J. Charity, Jack T. Harvey, Jannique G. Z. van Uffelen, Stuart J. H. Biddle, Rochelle M. Eime

**Affiliations:** 1 Institute for Resilient Regions, University of Southern Queensland, Springfield Campus, PO Box 4393, Raceview, QLD 4305 Australia; 2 Institute of Sport, Exercise and Active Living (ISEAL), Active Living & Public Health Program, Victoria University, Melbourne, VIC Australia; 3 Faculty of Health, Federation University Australia, Ballarat, VIC Australia; 4 Department of Kinesiology, Physical Activity, Sports and Health Research Group, KU Leuven - University of Leuven, B-3000 Leuven, Belgium

**Keywords:** Holistic, Mind-body, Participation prevalence, Physical activity surveillance, Yoga, Tai chi, Qigong, Pilates

## Abstract

**Background:**

In recent decades, the evidence supporting the physical and mental health benefits of holistic movement practices such as yoga and t’ai chi have become increasingly established. Consequently, investigating the participation prevalence and patterns of these practices is a relevant pursuit in the public health field. Few studies have provided population-level assessment of participation rates, however, and even fewer have focused on patterns over time. The purpose of this study was to examine participation prevalence and trends in yoga/Pilates and t’ai chi/qigong over a ten-year period in a nationally representative sample of Australians aged 15 years and over, with particular attention to sex and age. A secondary purpose was to juxtapose these findings with participation trends in traditional fitness activities over the same period.

**Methods:**

Data comprised modes and types of physical activity, age, and sex variables collected through the Exercise, Recreation and Sport Survey (ERASS), a series of independent cross-sectional Australia-wide surveys conducted yearly between 2001 and 2010. For each year, weighted population estimates were calculated for those participating in yoga/Pilates, t’ai chi/qigong, and fitness activities (e.g. aerobics, calisthenics). Linear regression and multiple logistic regression analyses were used to examine trends in prevalence rates over time and differences among sex and age (15–34; 35–54; 55+ years) groups, respectively.

**Results:**

Average prevalence rates between 2001 and 2010 were 3.0% (95% CI 2.9–3.1) for yoga/Pilates, 0.6% (95% CI 0.5–0.6) for t’ai chi/qigong, and 19.2% (95% CI 18.9–19.4) for fitness activities. Across the decade, overall participation rates remained relatively stable for yoga/Pilates and t’ai chi/qigong, while increasing linearly for fitness activities. For both genders and in all three age groups, participation in fitness activities increased, whereas only in the 55+ age group was there a significant increase in yoga/Pilates participation; participation in t’ai chi/qigong declined significantly in the two younger age groups.

**Conclusions:**

Participation rates in yoga/Pilates and t’ai chi/qigong in Australia were low and relatively stable. As fitness activities increased in popularity across the decade, holistic movement practices did not. These findings point to the need to investigate activity-specific barriers and facilitators to participation, including intrapersonal, interpersonal, organisational, and environmental factors.

## Background

The health benefits of physical activity have long been recognised [1]. Regular physical activity reduces the risk of chronic diseases (e.g. cardiovascular disease, diabetes, cancer) and poor mental health outcomes (e.g. depression), and improves/maintains functional status [1, 2]. There are many modes and types of physical activity, and present-day Western society offers a wide range of physical practices that can help people reap these health benefits. Some of the physical practices that have appeared and grown within Western cultures during the last century are holistic in nature [3, 4]. Sometimes referred to as ‘mind-body’ disciplines, these “holistic movement practices” (HMPs) are non-competitive and non-performance oriented in nature, and tend to go beyond the emphasis on health-related physical fitness by embedding the physical practice in an integrative philosophy concerned with holistic well-being. This can frequently include an emphasis on somatic experiencing, mindfulness, and/or personal growth. The most well-known of these practices are yoga and t’ai chi which are embedded in centuries-old non-western philosophies, but other practices embedded in more modern philosophies also exist, such as some forms of dance [5]. In the present study the focus is on yoga, Pilates, t’ai chi and qigong.

Yoga is an ancient holistic practice embedded in a philosophy that values moral, physical, mental, and ultimately spiritual, training [4, 6, 7]. Originating in India, yoga started to expand into Western cultures by the late nineteenth century [4]. Currently, there are many styles of yoga, comprising an extensive assortment of static and dynamic postures (called “asanas”), combined in various ways with concentration, breath regulation, and meditation. Yoga exercises are typically of light to moderate intensity (2.0–4.0 METs^1^ [8, 9]) and involve stretching and strengthening elements targeting a wide range of major and minor muscle groups [10]. Styles differ in their relative emphasis on physical intensity, flow, breathing techniques and meditation elements.

Pilates has a much shorter history than yoga, having been developed in the first half of the twentieth century by Joseph Pilates [11]. Pilates has been defined as “A mind-body exercise that requires core stability, strength, and flexibility, and attention to muscle control, posture, and breathing” [12] (p. 258). There is a strong emphasis on movement quality and learning to move with minimum effort, requiring the development of somatic awareness and elements of mindfulness [11, 13]. Moderate in intensity (3 METs [8, 9]), exercises are mat-based or involve the use of specialised equipment. There are also different styles within Pilates [11, 13], representing varying approaches to exercise format and pedagogical delivery.

Qigong and t’ai chi both have their origins in Chinese history and philosophy, dating back many centuries [14, 15], and are closely associated with concepts from traditional Chinese Medicine. These include the notion that the balancing of *yin* and *yang* (considered to be complementary opposing forces within the body, each associated with certain organs and qualities - such as inactivity-activity, decrease-increase, darkness-lightness) and the uninhibited flow of *qi* (considered to be a vital, life-force energy within the body) are essential for health and well-being [15, 16], and that this can be achieved through conscious movement and concentration. Qigong is older and more encompassing than t’ai chi [17]. T’ai chi can be seen as one expression of qigong, and tends to be more highly choreographed, lengthy and complex [17, 18]. Both practices include slow, meditative and flowing physical movements of mostly light intensity (1.5–3.0 METs [8, 9]), as well as purposeful regulation of breath and mind in coordination with those movements [17]. As with yoga, there are many styles of both t’ai chi and qigong [15, 19].

HMPs are increasingly being investigated for their health-related benefits, with respect to both physical and mental health, in clinical as well as non-clinical populations. Recent bibliometric reviews of yoga and t’ai chi, for example, show clearly increasing numbers of randomised controlled trials, particularly in the last decade [20, 21]. Growing numbers of systematic reviews report a wide range of health benefits associated with the various HMPs. Regular yoga practice has, for example, been found to: have clinically relevant positive effects on most risk factors for cardiovascular disease [22]; improve pulmonary function in healthy individuals [23]; produce beneficial changes in oxygen consumption and metabolism [24]; improve strength, flexibility, aerobic fitness, and physical and mental health in older adults [25]; and play a promising role in stress management [26]. Compared to various forms of aerobic exercise, yoga has been shown to be less beneficial for aerobic outcomes, but more beneficial for outcomes like balance, flexibility, total anti-oxidant status, salivary cortisol, heart rate variability, fatigue and stress [27]. Demonstrated effects of Pilates in healthy populations include improving flexibility, dynamic balance, and muscular endurance [28]. Research on health outcomes of t’ai chi has had a particularly strong focus on older adult populations. While t’ai chi and qigong have been found to be beneficial for improving balance, preventing falls, and reducing symptoms of depression and anxiety in general adult populations [29, 30], benefits for older adults include clinically relevant improvements in executive function in cognitively healthy adults, and attenuation of cognitive decline in general [31], improved static and dynamic balance and physical function, and reductions in fall risk, blood pressure, depression and anxiety [18, 32]. There is also some initial evidence that yoga, t’ai chi and qigong positively affect gene expression profiles in circulating immune cells, thereby affecting physiology “at its most fundamental level” [33] (p. 77).

While they clearly have their value as health-related exercise, HMPs also offer a holistic dimension that may have specific effects on their attractiveness as leisure or recreation pursuits. Participation motives for these practices often include motives specifically related to holistic benefits, for example, personal growth, somatic awareness, a felt sense of integrating body, mind and spirit, and enhanced spirituality [5, 34, 35]. HMPs may thus be pursued not only for their physical health benefits but also for their holistic effects. Experiencing these holistic benefits, however, may rely on a level of inner involvement and engagement with the self [36, 37] that is not typically needed in other forms of exercise, and this may potentially affect participation patterns.

With increasing appreciation of the potential health benefits of HMPs, investigating the participation prevalence and patterns of these practices becomes a relevant pursuit in the public health field. Population-level assessment of participation rates is not commonly reported, however, and participation trends over time even less so. An exception is the United States, where prevalence rates and trends in yoga, t’ai chi and qigong participation have been systematically surveyed via a Complementary and Alternative Medicine supplement added to the 2002, 2007, and 2012 versions of the National Health Interview Survey (NHIS), a nationally representative health surveillance system [38–40]. Based on a 12-month recall, reported participation rates for the three time points were 5.1%, 6.1%, and 9.5% for yoga; 1.3%, 1.0%, and 1.1% for t’ai chi; and 0.3%, 0.3%, and 0.3% for qigong, respectively. Another exception is England, where participation rates in yoga have been assessed through the national Health Survey for England (HSE) using a 4-week recall. Comparing data from 1997-99, 2003–4, and 2006 & 2008, Ding and Stamatakis [41] reported an increasing trend with participation rates of 0.46%, 0.94% and 1.11%, respectively. To our knowledge, the availability of trend data is currently limited to these two countries.

In this study, we draw on data collected through a national survey of leisure-time physical activity participation, the Exercise, Recreation and Sport Survey (ERASS), to examine participation prevalence and trends in several holistic movement practices over a 10-year period in Australia. Some relevant prevalence data for Australia have been provided in two earlier population-level studies. Analysing data from a nationally representative sample of 1067 adults aged 18+ years in 2005, Xue et al. [42] found a prevalence rate of 12.0% for yoga participation, and of 6.0% for combined participation in qigong, t’ai chi, and martial art. Drawing on nationally representative data from the Australian Longitudinal Study on Women’s Health (ALSWH) collected twice between 2006 and 2010, Bowe et al. [43] reported that the use of yoga and meditation (assessed using a single question) for health reasons remained more or less stable at 21%–22% for a younger (age 28–33 years in 2006; 31–36 years in 2009) cohort and at 18% for a mid-age (age 56–61 years in 2007; 59–64 years in 2010) cohort. These studies are restricted, however, by only reporting one point in time [42] or a focus on women and specific age cohorts only [43]. The ERASS survey used consistent measures to assess participation rates at yearly intervals, thus providing a useful basis for examining trends and serving as informative public health surveillance [44, 45].

It is difficult to gauge the significance of increases or decreases in HMPs without a wider appreciation of participation trends in other forms of physical activity. To help put the prevalence and trends of yoga/Pilates and t’ai chi/qigong participation in a wider context of participation in health-related exercise, we have therefore chosen to compare participation trends in these HMPs with participation trends in traditional fitness activities, such as aerobics and gym workouts. Fitness activities show some similarities to holistic movement practices in that they are generally non-competitive and non-performance-oriented in nature, and are often engaged in for health-related reasons [46]. They are different, however, in that they do not include the additional element of being embedded in a holistic philosophy. The comparison will thus give some context to the data on holistic movement practices, by allowing an impression of any growth in activities that show similarities but do not include the additional element of being embedded in a holistic philosophy. Because prevalence rates for yoga have been found to differ between men and women and among age groups [40, 47], and outcome research emphasizes the benefits of t’ai chi/qigong for older people in particular [18, 32], we chose to also examine prevalence and trends separately by sex and age.

The purpose of this study was to conduct a secondary data analysis of a nation-wide population survey to examine the participation prevalence and trends in yoga/Pilates and t’ai chi/qigong in Australia over the period 2001–2010, with particular attention to sex and age. An additional purpose was to compare these findings with participation trends in traditional fitness activities over the same period.

## Methods

### ERASS survey and sample

The Exercise, Recreation and Sport Survey (ERASS) entailed a series of independent cross-sectional national surveys conducted yearly between 2001 and 2010, with the aim of collecting information on the exercise-, recreation- and sport-related physical activities Australians participate in [48, 49]. The usefulness of ERASS for public health surveillance has been previously established [44], and various studies have drawn from ERASS data to describe, for example, participation trends of leisure-time physical activity [50], the diversity of physical activities engaged in by older people [51] and population percentages meeting muscle-strengthening activity guidelines [52].

The ERASS comprised a random survey stratified by state and territory, aimed at persons aged 15 years and over residing in occupied private dwellings. Using a computer-assisted telephone interview (CATI) system, data were collected quarterly, with households being sampled from the Electronic White Pages (2001–2006) or by Random Digit Dialling (2007–2010) [49]. On being contacted by telephone, respondents were informed about the purpose and background of the ERASS, assured of confidentiality of the data and were given the opportunity to ask questions. Verbal informed consent was indicated by the respondents’ willingness to participate in the telephone survey. De-identified annual data from 2001 to 2010 were analysed for this study. Ethics approval was granted by the Human Research Ethics Committee of the Federation University, Australia.

From 2001 to 2010, annual response rates were 49.0%, 48.0%, 45.3%, 41.0%, 34.0%, 42.0%, 31.4%, 25.7%, 25.2% and 23.1%, respectively. The decline in response rates reflects increased refusal rates [49], possibly attributable to a rise in commercial telemarketing calls making people less willing to participate, even in non-commercial surveys [42]. Sample sizes varied between 15,477 (2001) and 23,226 (2006), with a total number of 195,926 respondents over the 10 years. Averaged across the decade, 50.6% of respondents were female, 37.1% were aged 50 years and over, 29.6% had below high school education while 24.3% was university educated, 37.9% were in the two most disadvantaged quintiles of the ‘Index of Relative Socio-Economic Advantage and Disadvantage’ [53], 77.5% were from the states of New South Wales, Victoria or Queensland, and 68.4% were from metropolitan (rather than regional or remote) areas. Details of sample characteristics by year can be found in Bennie et al. [52]. Of the 195,926 people surveyed between 2001 and 2010, 159,293 (81.3%) engaged in some level of leisure-time physical activity during the 12 months preceding the date of interview.

### ERASS questionnaire

The ERASS questionnaire asked respondents about participation in leisure-time physical activity, defined as ‘*any physical activity done for exercise, recreation or sport in the past 12 months’*. Respondents were asked to exclude *‘any physical activity associated with work, household or garden chores’*. Those who indicated participation were asked to list the types of leisure-time physical activity undertaken. For each participant, a maximum of 10 activities was recorded. Activities were coded according to a predefined list including many sport activities and a range of non-sport leisure-time physical activities. A single code was assigned to yoga and Pilates, whilst t’ai chi and qigong were coded separately. An assortment of fitness activities were also coded separately. For the purpose of this study, we used the jointly coded *yoga/Pilates* variable, and combined t’ai chi, qigong, and Chinese exercises into a *t’ai chi/qigong* variable. For comparative purposes, a *fitness* variable was constructed by combining the following exercise activities: Calisthenics, Exercise Bike, Gymnasium Workouts, Military Exercise, Prime Movers (over 50s), Step Reebok, Aerobics, and Treadmill. The questionnaire also assessed a number of demographic variables. For this study we included sex and age.

### Statistical analysis

Analyses were conducted using the Complex Samples module of SPSS version 22 (SPSS Inc., Chicago, IL). To allow for valid population estimates, all data were weighted by state, region (metropolitan or rest of the state), age group, sex, and year, with weights based on Australian Bureau of Statistics (ABS) projections for persons in occupied private dwellings. For each year, percentages and 95% confidence intervals were calculated for the proportion of the population participating in yoga/Pilates, t’ai chi/qigong, and fitness activities. This was done for the total sample and by sex and age. Linear regression analysis was used to examine trends in prevalence rates over time, while chi-square tests of independence were used to examine differences between prevalence rates in sex and age categories for each year. The level of statistical significance was set at *p* < 0.05. To further examine categorical differences, multiple logistic regression analyses were employed for sex and age, using “male” and “15–34 years” as reference categories, respectively. In this analysis, mutual adjustments were made for sex and age, and year of study was included as a covariate to adjust for yearly variations.

## Results

### Overall

**Fig. 1:**
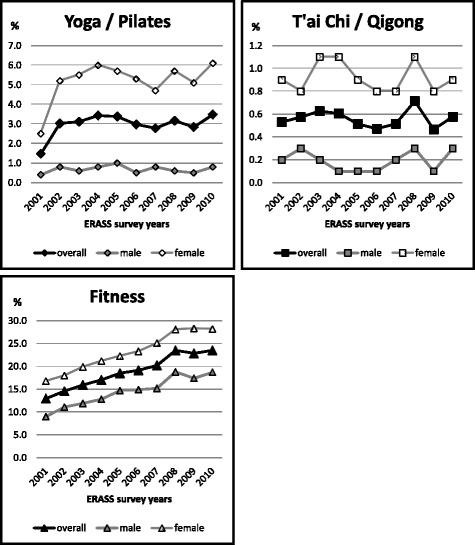
Ten-year participation trends in yoga/Pilates, t’ai chi/qigong and fitness, showing prevalence rates for all participants (“overall”) and separately for males and females

Weighted population estimates are reported in Table 1. Figure 1 shows yearly prevalence rates between 2001 and 2010, overall and by sex. For yoga/Pilates, overall prevalence rates ranged between 1.5% and 3.5% across the decade, with a mean of 3.0% (95% CI: 2.9–3.1). The lowest of these rates were reported in 2001 and the highest in 2010, and the rates doubled from 2001 to 2002. There was no significant linear trend over the 10 years. For t’ai chi/qigong, overall prevalence rates varied between 0.5% and 0.7%, with a mean of 0.6% (95% CI: 0.5–0.6). There was no significant linear trend, and participation rates were relatively stable over the decade. For fitness, overall prevalence rates ranged between 13.0% and 23.5%, with an average of 19.2% (95% CI: 18.9–19.4). There was a significant linear trend, showing a clear increase in participation over the ten years.

**Table 1:** Percentages of the Exercise, Recreation and Sport Survey (ERASS) 2001–2010 sample (*n* = 195, 926) reporting *any* participation in yoga/pilates, t’ai chi/qigong, and fitness activities during the past 12-months

	Prevalence of participation in the past 12 months^a^										
	2001	2002	2003	2004	2005	2006	2007	2008	2009	2010	Trend
	% (95% CI)										*p-value* ^*b*^
TOTAL SAMPLE											
Yoga/Pilates	1.5 (1.2–1.8)	3.0 (2.7–3.4)	3.1 (2.8–3.5)	3.4 (3.0–3.9)	3.4 (3.0–3.8)	3.0 (2.6–3.4)	2.8 (2.5–3.1)	3.2 (2.9–3.5)	2.8 (2.6–3.2)	3.5 (3.1–3.9)	0.175
T’ai chi/Qigong	0.5 (0.4–0.7)	0.6 (0.4–0.8)	0.6 (0.5–0.8)	0.6 (0.5–0.8)	0.5 (0.4–0.7)	0.5 (0.4–0.6)	0.5 (0.4–0.7)	0.7 (0.6–0.9)	0.5 (0.4–0.6)	0.6 (0.5–0.7)	0.887
Fitness	13.0 (12.2–13.7)	14.6 (13.8–15.4)	15.9 (15.2–16.8)	17.0 (16.2–17.9)	18.5 (17.6–19.4)	19.2 (18.3–20.1)	20.2 (19.4–21.0)	23.5 (22.7–24.4)	22.9 (22.0–23.7)	23.5 (22.7–24.4)	<0.001
BY SEX											
Yoga/Pilates											
Males	0.4 (0.2–0.7)	0.8 (0.6–1.2)	0.6 (0.4–1.0)	0.8 (0.6–1.2)	1.0 (0.7–1.5)	0.5 (0.3–0.8)	0.8 (0.6–1.1)	0.6 (0.4–0.8)	0.5 (0.4–0.8)	0.8 (0.6–1.1)	0.864
Females	2.5 (2.1–3.0)	5.2 (4.6–6.0)	5.5 (4.9–6.3)	6.0 (5.3–6.8)	5.7 (5.0–6.4)	5.3 (4.7–6.1)	4.7 (4.2–5.2)	5.7 (5.1–6.3)	5.1 (4.6–5.7)	6.1 (5.5–6.8)	0.146
*p-value* ^*c*^	<0.001	<0.001	<0.001	<0.001	<0.001	<0.001	<0.001	<0.001	<0.001	<0.001	
T’ai chi/Qigong											
Males	0.2 (0.1–0.3)	0.3 (0.2–0.6)	0.2 (0.1–0.4)	0.1 (0.1–0.3)	0.1 (0.1–0.3)	0.1 (0.1–0.3)	0.2 (0.1–0.4)	0.3 (0.2–0.5)	0.1 (0.0–0.2)	0.3 (0.2–0.4)	0.948
Females	0.9 (0.7–1.2)	0.8 (0.6–1.1)	1.1 (0.8–1.4)	1.1 (0.8–1.4)	0.9 (0.7–1.2)	0.8 (0.6–1.1)	0.8 (0.6–1.1)	1.1 (0.9–1.4)	0.8 (0.7–1.0)	0.9 (0.7–1.1)	0.803
*p-value* ^*c*^	<0.001	0.008	<0.001	<0.001	<0.001	<0.001	<0.001	<0.001	<0.001	<0.001	
Fitness											
Males	9.0 (8.1–10.0)	11.1 (10.1–12.2)	11.9 (10.9–13.0)	12.8 (11.7–14.0)	14.7 (13.5–15.9)	14.9 (13.7–16.1)	15.2 (14.1–16.3)	18.8 (17.6–20.0)	17.4 (16.2–18.6)	18.7 (17.5–19.9)	<0.001
Females	16.8 (15.7–18.0)	18.0 (16.9–19.2)	19.9 (18.8–21.1)	21.2 (20.0–22.5)	22.3 (21.1–23.5)	23.3 (22.1–24.6)	25.1 (24.0–26.2)	28.1 (26.9–29.3)	28.3 (27.1–29.5)	28.2 (27.0–29.5)	<0.001
*p-value* ^*c*^	<0.001	<0.001	<0.001	<0.001	<0.001	<0.001	<0.001	<0.001	<0.001	<0.001	
BY AGE											
Yoga/Pilates											
15–34 years	2.0 (1.5–2.6)	3.5 (2.8–4.3)	3.6 (2.9–4.4)	3.4 (2.7–4.3)	3.0 (2.4–3.8)	2.7 (2.1–3.5)	2.6 (2.0–3.2)	3.4 (2.8–4.1)	2.7 (2.1–3.3)	3.7 (3.0–4.6)	0.590
35–54 years	1.5 (1.2–2.0)	3.5 (3.5–2.9)	3.4 (2.8–4.1)	4.5 (3.8–5.3)	4.4 (3.7–5.2)	3.5 (3.0–4.2)	3.5 (3.0–4.1)	4.0 (3.4–4.7)	3.4 (2.9–4.0)	4.0 (3.4–4.6)	0.182
55+ years	0.7 (0.5–1.1)	1.8 (1.3–2.3)	2.1 (1.6–2.7)	2.0 (1.5–2.6)	2.5 (2.0–3.2)	2.6 (2.1–3.2)	2.1 (1.7–2.6)	1.8 (1.4–2.3)	2.4 (2.0–2.8)	2.7 (2.3–3.2)	0.028
*p-value* ^*c*^	0.001	<0.001	0.004	<0.001	<0.001	0.080	0.001	<0.001	0.020	0.020	
T’ai chi/Qigong											
15–34 years	0.1 (0.0–0.4)	0.3 (0.2–0.6)	0.2 (0.1–0.4)	0.2 (0.1–0.5)	0.1 (0.0–0.2)	0.2 (0.1–0.6)	0.1 (0.0–0.3)	0.2 (0.1–0.4)	0.0 (0.0–0.1)	0.0 (0.0–0.1)	0.025
35–54 years	0.5 (0.3–0.7)	0.5 (0.3–0.8)	0.5 (0.3–0.8)	0.6 (0.4–0.9)	0.4 (0.2–0.6)	0.2 (0.1–0.4)	0.3 (0.1–0.4)	0.5 (0.3–0.7)	0.2 (0.1–0.4)	0.3 (0.2–0.6)	0.047
55+ years	1.2 (0.8–1.7)	1.0 (0.7–1.4)	1.4 (1.4–1.9)	1.2 (0.9–1.7)	1.2 (0.9–1.7)	1.1 (0.9–1.5)	1.3 (1.0–1.7)	1.7 (1.3–2.2)	1.3 (1.0–1.6)	1.4 (1.1–1.8)	0.092
*p-value* ^*c*^	<0.001	0.008	<0.001	<0.001	<0.001	<0.001	<0.001	<0.001	<0.001	<0.001	
Fitness											
15–34 years	17.0 (15.6–18.6)	19.7 (18.1–21.3)	19.6 (18.1–21.2)	22.8 (21.1–24.6)	24.0 (22.3–25.8)	23.4 (21.6–25.3)	23.7 (22.1–25.3)	27.3 (25.7–29.1)	27.2 (25.4–29.1)	27.2 (25.3–29.1)	<0.001
35–54 years	12.2 (11.1–13.4)	13.9 (12.7–15.1)	15.1 (13.8–16.4)	14.9 (13.7–16.2)	17.9 (16.6–19.3)	19.6 (18.2–21.0)	21.1 (19.9–22.4)	24.4 (23.1–25.8)	24.0 (22.8–25.4)	25.1 (23.8–26.5)	<0.001
55+ years	8.1 (7.1–9.2)	8.8 (7.8–10.0)	12.3 (11.0–13.6)	12.4 (11.2–13.6)	12.3 (11.1–13.5)	13.7 (12.5–15.0)	15.2 (14.0–16.4)	18.0 (16.7–19.3)	16.6 (15.5–17.7)	17.7 (16.7–18.8)	<0.001
*p-value* ^*c*^	<0.001	<0.001	<0.001	<0.001	<0.001	<0.001	<0.001	<0.001	<0.001	<0.001	

^a^Prevalence of respondents who reported participating at least once in the last 12 months

^b^ *p*-value for linear regression analysis over time (2001–2010)

^c^ *p*-value for chi-square test of the difference between categories

### Sex

With respect to sex, participation rates for all three activities were significantly higher among women than among men, in every year between 2001 and 2010. This difference was largest for yoga/Pilates, where the odds of women participating were almost 8 times (OR = 7.93, 95% CI: 6.97–9.03) higher than of men participating, while for t’ai chi/qigong and fitness, these odds ratios were 4.6 (95% CI: 3.62–5.77) and 1.8 (95% CI: 1.75–1.89), respectively (Table 2). Consistent with the overall prevalence trends, there were significant linear trends for fitness for both sexes but not for yoga/Pilates or t’ai chi/qigong for either of the sexes (Table 1).

**Table 2:** Adjusted^a^ odds ratios (OR), and their 95% confidence intervals (95% CI), of any participation in yoga/Pilates, t’ai chi/qigong, and fitness in the past 12 months

	Yoga/Pilates in the past 12 months	T’ai chi/Qigong in the past 12 months	Fitness in the past 12 months
	OR	OR	OR
Sex (ref: male)			
Females	7.93 (6.97–9.03)	4.57 (3.62–5.77)	1.82 (1.75–1.89
*p-value* ^*b*^	*<0.001*	*<0.001*	*<0.001*
Age (ref: 15–34 years)			
35–54 years	1.19 (1.08–1.30)	2.66 (1.84–3.84)	0.77 (0.74–0.80)
55+ years	0.67 (0.60–0.74)	9.03(6.43–12.69)	0.51(0.49–0.53)
*p-value* ^*b*^	*<0.001*	*<0.001*	*<0.001*

^a^Adjusted for year and for the other explanatory variables in the model

^b^ *p*-value based on the likelihood ratio Wald chi-square test

### Age

**Fig. 2:**
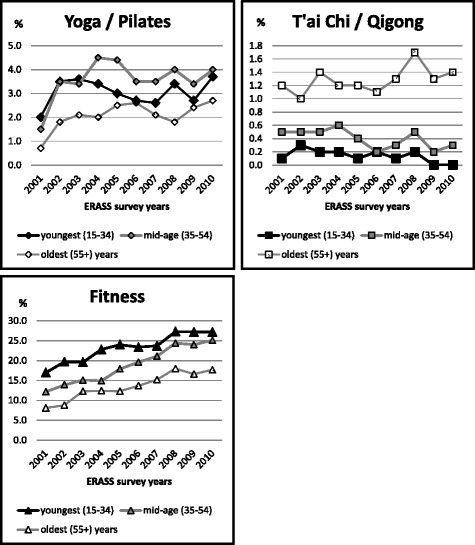
Ten-year participation trends in yoga/Pilates, t’ai chi/qigong and fitness, showing prevalence by age groups

With respect to age, the three activities showed differential patterns (Tables 1 and 2; Fig. 2). Significant differences in participation rates among the age categories were found for each year and each activity except for yoga/Pilates in 2006, when the differences in participation rates between the three age groups did not reach the level of statistical significance.

For yoga/Pilates, the mid-age (35–54 years) group, although starting out with lower participation rates than the youngest (15–34 years) age group, tended to have higher participation rates, particularly from 2004 onwards (Table 1). The likelihood of participation was somewhat higher (OR = 1.19, 95% CI: 1.08–1.30) for this group than the youngest age group (Table 2). The oldest (55+ years) age group had the lowest likelihood of participation (OR = 0.67, 95% CI: 0.60–0.74). However, this age group was the only one that showed a significant linear trend of participation rates across the decade, increasing from 0.7% in 2001 to 2.7% in 2010 (Table 1).

For t’ai chi/qigong, the highest participation rate was in the oldest (55+) age group (Table 2). This age group had more than 9 times higher odds of participation in t’ai chi/qigong than the youngest age group (OR = 9.03, 95% CI: 6.43–12.69). There was no linear trend across the years for this age group. There were significant linear trends for the two younger age groups, both suggesting a decreasing trend in participation levels across the decade (Table 1).

For fitness activities, the highest participation rate was in the youngest (15–34) age group (Table 2), with likelihood of participation decreasing significantly with increasing age. All three age groups showed similar significant linear increases in participation rates across the decade (Table 1).

Thus, differential patterns are evident for the three activities when taking into account age. For fitness activities, participation rates tended to decline from younger to older age groups, while for t’ai chi/qigong the reverse was true, and for yoga/Pilates, participation rates tended to be highest for the mid age group. Visual inspection (Table 1; Fig. 2) suggests that within each age group, however, participation prevalence was similarly distributed to those for the overall figures, in that the highest average participation rates were for fitness activities, followed at a considerable distance by yoga/Pilates, with t’ai chi/qigong being the lowest.

## Discussion

This is the first study to report participation trends of several holistic movement practices in Australia. We investigated the prevalence and trends of yoga/Pilates and t’ai chi/qigong in Australia during the 2001–2010 decade, and compared these to the prevalence and trends of fitness activities over the same period.

Results showed that while there was a significant linear increase in the percentage of Australians participating in fitness activities, overall participation rates in yoga/Pilates and in t’ai chi/qigong remained relatively stable across the decade. Moreover, fitness participation rates increased significantly in all subgroups of sex and age, while a significant increase in yoga/Pilates participation was only found for the 55+ age group. Furthermore, a small but significant decline in t’ai chi/qigong participation was found for the 15–34 and 35–54 age groups. Considering these trends, it might be that public health messages about the health benefits of physical activity have had an impact on the uptake of fitness activities in general, but less so on the uptake of holistic movement practices like yoga/Pilates and t’ai chi/qigong.

While there appears to have been a brief “boom” in yoga/Pilates participation between 2001 and 2002 with proportions doubling overall and in virtually all subgroups of sex and age, this did not continue over the decade. Lacking comparable data from before 2001, it is difficult to say whether the “boom” was a brief blip or the end of a longer period of growth. As the ERASS was discontinued after 2010, no equivalent data are available since then to check whether the increase between 2009 and 2010 constituted a mere fluctuation or the beginning of a period of growth.

### Comparisons

Direct comparisons of prevalence rates with those reported in other studies are problematic due to variations in question format (e.g. open-ended or prompted), recall period (e.g. 12 months vs 4 weeks), survey mode (face-to-face, telephone, or internet), and context (e.g., general health or complementary and alternative medicine, vs exercise, sport and recreation). Nevertheless, a rough comparison of trends may be possible in some cases.

Across approximately overlapping periods, there were significant increases in levels of yoga participation in both the United States (comparing 2002, 2007, and 2012 data [40]) and England (comparing 1997/8 and 2006/8 data [41]). This growth contrasts with the lack of increase in yoga/Pilates participation in Australia between 2001 and 2010. Although for women only and using a combined yoga/meditation question, Bowe et al. [43] also noted a stabilization of prevalence rates in Australia between 2006 and 2009, suggesting a possible cultural difference between the countries in the receptiveness to and/or marketing of yoga and related practices. Participation trends for t’ai chi/qigong in the United States over the 2002–2007-2012 period, on the other hand, showed a similar lack of change to Australia, with t’ai chi and qigong likewise having lower prevalence rates than yoga [40]. There is scope for future studies to investigate the socio-cultural and environmental aspects that may have contributed to these differences and similarities. Knowledge about social and environmental infrastructures that appear to facilitate growth might then inform attempts at increasing participation in practices and areas with currently stagnant or declining participation rates.

One socio-cultural issue that deserves attention is ethnicity. Australia is a culturally and linguistically diverse country, its population shaped and reshaped over many years of migration [54]. Between 2001 and 2010 the Australian population grew by 14.3%, an increase that was in large part due to immigration [55]. A substantial and increasing number of immigrants during this period came from India and China, the “home” countries of yoga and t’ai chi/qigong [56]. In the United States, Birdee et al. [57] reported a significantly higher proportion of Asian residents among t’ai chi/qigong users than among non-t’ai chi/qigong users, suggesting that there could be a cultural “match” that might make these practices more appealing, and possibly easier to access, to those from a similar culture. However, in Australia, the substantial influx of Chinese and Indian immigrants in the 2001–2010 decade was not reflected in increased participation rates in yoga/Pilates and t’ai chi/qigong. Given these findings, and Australia’s multi-cultural make-up in general, investigating ethnicity-specific barriers and facilitators could be one avenue of socio-cultural future research. Ethnicity was only assessed in the last three ERASS surveys, thus we were not able to include it in our trends analyses.

### Sex

An interesting observation is that at every point in time and for all three practices, participation rates were higher among women than men, although ratios differed between the practices, with the differences most substantial for yoga/Pilates. A predominance of female participants is a common finding for yoga [41, 47, 58]. Few studies have looked at t’ai chi/qigong or fitness-related sex differences at a population level, but those that did reported no sex differences for participation in t’ai chi/qigong in the United States [57] or fitness-related activities in England [59], suggesting there may be a culture-related aspect, perhaps unique to Australia, to this finding. Despite the differences between prevalence rates, participation trends showed similar patterns for both females and males across the decade, implying that whatever factors were responsible for the stagnant levels of participation in HMPs, the effects may have been similar for men and women. Future research needs to investigate sex-specific facilitators and barriers to participation in HMPs, with concomitant attention to local and cultural norms and expectations.

### Age

It is noteworthy that among the oldest age group (55+ years), participation rates for yoga/Pilates increased significantly over the 10-year survey period, while there was no similar growth in participation rates for t’ai chi/qigong. While this is promising with respect to the uptake of yoga/Pilates among older adults, it is somewhat surprising given the heavy research emphasis on t’ai chi/qigong benefits for older people [18, 31, 32]. It is difficult to say what may have caused this difference. Lack of familiarity with the benefits of t’ai chi has been cited as a primary barrier to participation among older non-t’ai chi participants [60]. This may have played a role, but it is plausible that a range of factors may be involved, varying from marketing, to availability of qualified teachers, to social-cultural and geographical/environmental infrastructures.

While not increasing, it was evident that among t’ai chi/qigong practitioners, the higher participation levels were in the oldest age group, which is fitting with the research outcomes that emphasize positive health effects for older adults; meanwhile, participation levels in both younger age groups declined over the decade. It may be that an unfortunate consequence of increasing emphasis on the benefits of t’ai chi/qigong for older people is that perceptions of t’ai chi/qigong become associated with older age, thereby becoming less attractive to those under 55. This would be regrettable, as t’ai chi/qigong also offer various benefits for younger age groups, in particular in dealing with the pressures of modern life, including ameliorating effects on stress, anxiety and depression [29, 30, 61], and potentially boosting immune function and cardiorespiratory fitness [62, 63]. Furthermore, early research suggests long-term practice of t’ai chi/qigong may help slow down age-related cognitive decline [64], and early uptake might support the accumulation of these effects. It has also been argued [65] that t’ai chi could be useful for young people in enabling them to train mind-body awareness and control, as well as relaxation, thus establishing important health-related life skills.

### Barriers

Taking up and sustaining participation in physical activities is challenging for many people. Ecological models specify that health behaviours are influenced by factors at multiple levels, including intra- and inter-personal, organizational, community, and public policy levels, each of which can be affected by sociocultural and physical environmental factors [66].

Non-holistic health and fitness oriented activities were shown in our analyses to increase in popularity, while HMPs were not. What could explain this discrepancy? Reasons might be related to such factors as image, marketing, and/or availability. It is also possible that the holistic embeddedness could act as a barrier to some populations; for example, negative preconceptions of yoga as being associated with “alternative” lifestyles or being some type of religion have been cited as barriers to participation by non-participants [67]. We know little about the content and extent of marketing messages and strategies used in the promotion of HMPs, or about which population groups are targeted. Neither do we know much about the extent to which the delivery of the philosophical embeddedness of holistic movement practices has been adapted to suit different population groups. Additionally, we have limited systematic knowledge about the social and environmental infrastructures through which these practices are on offer for the different population groups. Another aspect that might be related to the discrepancy is the fact that HMPs call for a level of inner involvement [36, 37] that is generally absent in non-holistic health and fitness activities, and we have little knowledge about the role that this aspect plays as either an attractor or barrier to participation. There is ample scope for research to address the above factors and investigate their role in facilitating or constraining HMP participation.

### Limitations and strengths

This study has several limitations. First, yoga and Pilates were assessed as a joint activity. While they share various similarities (e.g., attention to flexibility, muscle control, breathing, mind-body integration [12, 58]), there are also substantial differences, in particular in underlying philosophy. This was an inherent limitation in the data set, and suggests a lack of appreciation of the distinction between the two practices within the sport-orientated context in which the survey was designed. Regrettably, it means it is not possible to draw conclusions about the participation trends for each of the two practices separately. These practices deserve to be treated individually in future surveillance studies. Second, because the data relied on self-report measures they may include a recall and/or response bias. However, this is the case for most surveillance studies examining participation in specific activities. It may also be argued that people who are physically active may have been more inclined to respond to a survey on sport, exercise and recreation participation, which may have introduced an over-estimation bias. On the other hand, using an open-ended rather than prompted question and the use of a 12-month recall may have led to people reporting more habitual activities [44]. Third, though the threshold of “at least once in the previous 12 months” is not uncommon in surveillance studies [40, 50, 57, 68], a more stringent definition of participation might have led to different results. Future studies may address this issue. Lastly, the results are specific to the Australian situation. Generalizability to other countries will likely depend on similarities in socio-cultural aspects, population profiles, and social-political-geographical infrastructures that determine the availability and accessibility of HMPs. Nevertheless, the results of the current study point to aspects that may be of relevance to other countries, such as a possible discrepancy in growth rates between HMPs and general fitness activities, and a prompt to investigate the cultural and infrastructural factors that may explain these.

Strengths of this study include the use of large broadly representative national samples [50], quarterly data collection minimising seasonality bias, and the repetition of the same survey questions over multiple years [45].

## Conclusion

Nationally representative studies documenting participation trends in HMPs over a number of years have thus far been limited, with data available only for the USA [40] and England [41]. This study added to the knowledge of participation trends in HMPs by providing data from a different country, Australia, and by comparing the trends in HMP participation to those in fitness activities over the same 10-year period. Results showed that, while participation rates in yoga/Pilates tended to be higher than in t’ai chi/qigong among Australians between 2001 and 2010, for both types of practices rates remained relatively level over this period. Compared to fitness activities, which saw a linear increase across the decade, this suggests that holistic movement practices became a relatively less popular choice among those choosing new or additional modes of physical activity. This points to the need to further investigate not only individual-level facilitators and barriers to participation in holistic movement practices, but also the social, commercial, organizational and environmental infrastructures through which these practices are offered. Such a closer look at cultural as well as infrastructural factors may also elucidate possible reasons for participation differences between countries. In terms of prevalence, it must be noted that the great majority of Australians do *not* participate in holistic movement practices, or even in fitness activities (although the increasing trend in fitness participation across the 2001–2010 decade is promising). There is thus, even among groups that are currently most represented, substantial room to grow participation rates in all three types of activity.

## Abbreviations

ERASS: Exercise, Recreation and Sport Survey
HMP: Holistic movement practice
MET: metabolic equivalent

## Footnotes

1 MET = metabolic equivalent, the amount of oxygen consumed while sitting at rest. One MET is equal to 3.5 ml O_2_ per kg body weight x min [69].

## References

[1] Lee IM, Shiroma EJ, Lobelo F, Puska P, Blair SN, Katzmarzyk PT (2012). Effect of physical inactivity on major non-communicable diseases worldwide: an analysis of burden of disease and life expectancy. Lancet.

[2] Warburton DER, Nicol CW, Bredin SSD (2006). Health benefits of physical activity: the evidence. Can Med Assoc J.

[3] Brown D, Leledaki A (2010). Eastern movement forms as body-self transforming cultural practices in the west: towards a sociological perspective. Cult Sociol.

[4] DeMichelis E (2004). A history of modern yoga: Patanjali and western esotericism.

[5] Vergeer I. Participation motives for a holistic dance-movement practice. Int J Sport Exercise Psy. 2016. doi:10.1080/1612197X.2016.1167759.

[6] Alter J (2006). Yoga at the fin de Siècle : muscular Christianity with a ‘Hindu’ twist. Int J Hist Sport.

[7] Smith BR (2007). Body, mind and Spirit? Towards an analysis of the practice of yoga. Body Soc.

[8] Ainsworth BE, Haskell WL, Herrmann SD, Meckes N, Bassett DR, Tudor-Locke C (2011). The compendium of physical activities tracking guide.

[9] Ainsworth BE, Haskell WL, Herrmann SD, Meckes N, Bassett DR, Tudor-Locke C (2011). 2011 compendium of physical activities: a second update of codes and MET values. Med Sci Sport Exer.

[10] Pate JL, Buono MJ (2014). The physiological responses to Bikram yoga in novice and experienced practitioners. Altern Ther Health Med.

[11] Latey P. The Pilates method: history and philosophy. J Bodywork Mov Ther. 2001;5(4):275–82 8p.

[12] Wells C, Kolt GS, Bialocerkowski A (2012). Defining Pilates exercise: a systematic review. Complement Ther Med..

[13] Adams M, Caldwell K, Atkins L, Quin R (2012). Pilates and mindfulness: a qualitative study. J Dance Educ.

[14] Deadman P. A Brief History of Qigong. J Chinese Med. 2014(105):5–17 3p.

[15] Ospina MB, Bond K, Karkhaneh M, Tjosvold L, Vandermeer B, Liang Y (2007). Meditation practices for health: state of the research. Evidence report/technology assessment.

[16] Li G, Yuan H, Zhang W (2014). Effects of tai chi on health related quality of life in patients with chronic conditions: a systematic review of randomized controlled trials. Complement Ther Med..

[17] Jahnke R, Larkey L, Rogers C, Etnier J, Lin F (2010). A comprehensive review of health benefits of qigong and tai chi. Am J Health Promot.

[18] Rogers CE, Larkey LK, Keller C (2009). A review of clinical trials of tai chi and qigong in older adults. Western J Nurs Res.

[19] Guo Y, Qiu P, Liu T (2014). Tai Ji Quan: an overview of its history, health benefits, and cultural value. J Sport Health Sci.

[20] Cramer H, Lauche R, Dobos G. Characteristics of randomized controlled trials of yoga: a bibliometric analysis. BMC Complem Altern M. 2014;14 10.1186/1472-6882-14-328 PMC4161862 25183419

[21] Yang GY, Wang LQ, Ren J, Zhang Y, Li ML, Zhu YT, et al. Evidence base of clinical studies on Tai Chi: A bibliometric analysis. PLoS One. 2015;10(3). 10.1371/journal.pone.0120655 PMC4361587 25775125

[22] Cramer H, Lauche R, Haller H, Steckhan N, Michalsen A, Dobos G (2014). Effects of yoga on cardiovascular disease risk factors: a systematic review and meta-analysis. Int J Cardiol.

[23] Abel AN, Lloyd LK, Williams JS (2013). The effects of regular yoga practice on pulmonary function in healthy individuals: a literature review. J Altern Complem Med..

[24] Tyagi A, Cohen M (2013). Oxygen consumption changes with yoga practices: a systematic review. J Evid Based Complementary Altern Med..

[25] Patel NK, Newstead AH, Ferrer RL (2012). The effects of yoga on physical functioning and health related quality of life in older adults: a systematic review and meta-analysis. J Altern Complem Med..

[26] Sharma M (2014). Yoga as an alternative and complementary Approach for stress management: a systematic review. J Evid Based Complementary Altern Med.

[27] Ross A, Thomas S (2010). The health benefits of yoga and exercise: a review of comparison studies. J Altern Complem Med..

[28] Cruz-Ferreira A, Fernandes J, Laranjo L, Bernardo LM, Silva A (2011). A systematic review of the effects of pilates method of exercise in healthy people. Arch Phys Med Rehab.

[29] Lee MS, Ernst E (2012). Systematic reviews of t'ai chi: an overview. Brit J Sport Med.

[30] Yin J, Dishman RK (2014). The effect of tai chi and Qigong practice on depression and anxiety symptoms: a systematic review and meta-regression analysis of randomized controlled trials. Ment Health Phys Act.

[31] Wayne PM, Walsh JN, Taylor-Piliae RE, Wells RE, Papp KV, Donovan NJ (2014). Effect of tai chi on cognitive performance in older adults: systematic review and meta-analysis. J Am Ger Soc.

[32] Jiménez-Martín PJ, Meléndez-Ortega A, Albers U, Schofield D (2013). Review article: a review of tai chi Chuan and parameters related to balance. Eur J Integr Med.

[33] Saatcioglu F (2013). Regulation of gene expression by yoga, meditation and related practices: a review of recent studies. Asian J Psychiat.

[34] Park CL, Riley KE, Bedesin E, Stewart VM. Why practice yoga? Practitioners’ motivations for adopting and maintaining yoga practice. J Health Psychol. 2016;21(6):887–96 10p. 10.1177/1359105314541314 25030795

[35] Yang Y, Decelle S, Reed M, Rosengren K, Schlagal R, Greene J. Subjective experiences of older adults practicing Taiji and qigong. J Aging Res. 2011; doi:10.4061/2011/650210. 10.4061/2011/650210 PMC3134827 21773028

[36] Büssing A, Edelhäuser F, Weisskircher A, Fouladbakhsh JM, Heusser P. Inner correspondence and peacefulness with practices among participants in Eurythmy therapy and yoga: a validation study. Evid Based Complement Alternat Med. 2011. doi:10.1155/2011/329023. 10.1155/2011/329023 PMC2952301 20953427

[37] Wayne PM, Kaptchuk TJ (2008). Challenges inherent to T’ai chi research: part I – T’ai chi as a complex multicomponent intervention. J Altern Complem Med.

[38] Barnes PM, Powell-Griner E, McFann K, Nahin RL (2004). Complementary and alternative medicine use among adults: United States, 2002. Semin Integr Med.

[39] Barnes PM, Bloom B, Nahin RL (2008). Complementary and alternative medicine use among adults and children: United States, 2007.

[40] Clarke TC, Black LI, Stussman BJ, Barnes PM, Nahin RL (2015). Trends in the use of complementary health approaches among adults: United States, 2002–2012.

[41] Ding D, Stamatakis E. Yoga practice in England 1997-2008: Prevalence, temporal trends, and correlates of participation. BMC Res Notes. 2014:7(1). 10.1186/1756-0500-7-172 PMC3987846 24661723

[42] Xue CCL, Zhang AL, Lin V, Da Costa C, Story DF (2007). Complementary and alternative medicine use in Australia: a national population-based survey. J Altern Complem Med..

[43] Bowe S, Adams J, Lui C-W, Sibbritt D (2015). A longitudinal analysis of self-prescribed complementary and alternative medicine use by a nationally representative sample of 19,783 Australian women, 2006–2010. Complement Ther Med.

[44] Merom D, Bauman A, Ford I (2004). The public health usefulness of the exercise recreation and sport survey (ERASS) surveillance system. J Sci Med Sport.

[45] Veal AJ (2003). Tracking change: leisure participation and policy in Australia, 1985-2002. Ann Leisure Res.

[46] Ingledew DK, Markland D (2008). The role of motives in exercise participation. Psychol Health.

[47] Cramer H, Ward L, Steel A, Lauche R, Dobos G, Zhang Y (2016). Prevalence, patterns, and predictors of yoga use: results of a U.S. nationally representative survey. Am J Prev Med.

[48] Australian Sports Commission. Recreation and Sport Survey (ERASS): Participation in Exercise Recreation and Sport - Annual Report 2010. 2010. http://www.ausport.gov.au/information/casro/ERASS. Accessed 9 Dec 2014.

[49] Australian Sports Commission. Recreation and Sport Survey (ERASS): Participation in Exercise Recreation and Sport - Methodology Report 2010. 2010. http://www.ausport.gov.au/information/casro/ERASS. Accessed 9 Dec 2014.

[50] Eime RM, Sawyer N, Harvey JT, Casey MM, Westerbeek H, Payne WR (2015). Integrating public health and sport management: sport participation trends 2001-2010. Sport Manag Rev.

[51] Merom D, Cosgrove C, Venugopal K, Bauman A (2012). Original research: how diverse was the leisure time physical activity of older Australians over the past decade?. J Sci Med Sport.

[52] Bennie JA, Pedisic Z, Van Uffelen JGZ, Charity MJ, Harvey JT, Banting LK (2016). Pumping iron in Australia: prevalence, trends and sociodemographic correlates of muscle strengthening activity participation from a national sample of 195,926 adults. PLoS One.

[53] Australian Bureau of Statistics. What is SEIFA? 2013. http://www.abs.gov.au/ausstats/abs@.nsf/Lookup/by%20Subject/2033.0.55.001~2011~Main%20Features~What%20is%20SEIFA%3f~4. Accessed 2 Aug 2016.

[54] Australian Bureau of Statistics. Australia’s population by country of birth. 2016. http://www.abs.gov.au/ausstats/abs@.nsf/Latestproducts/3412.0Main%20Features32015-16?opendocument&tabname=Summary&prodno=3412.0&issue=2015-16&num=&view=. Accessed 2 Aug 2016.

[55] Australian Bureau of Statistics. Australian historical population statistics - Population size and growth. 2014. http://www.abs.gov.au/AUSSTATS/abs@.nsf/DetailsPage/3105.0.65.0012014?OpenDocument. Accessed 2 Aug 2016.

[56] Australian Government (2013). Australia’s migration trends 2011–12.

[57] Birdee GS, Wayne PM, Davis RB, Phillips RS, Yeh GY (2009). T'ai chi and Qigong for health: patterns of use in the United States. J Altern Complem Med..

[58] Park CL, Braun T, Siegel T (2015). Who practices yoga? A systematic review of demographic, health-related, and psychosocial factors associated with yoga practice. J Behav Med.

[59] Stamatakis E, Chaudhury M (2008). Temporal trends in adults' sports participation patterns in England between 1997 and 2006: the health survey for England. Brit J Sport Med..

[60] Gryffin PA, Chen WC, Chaney BH, Dodd VJ, Roberts B (2015). Facilitators and barriers to tai chi in an older adult community: a theory-driven Approach. Am J Health Educ.

[61] Wang C, Bannuru R, Ramel J, Kupelnick B, Scott T, Schmid CH (2010). Tai Chi on psychological well-being: systematic review and meta-analysis. BMC Complem Altern M.

[62] Ho RTH, Wang C-W, Ng S-M, Ho AHY, Ziea ETC, Wong VT, et al. The Effect of T'ai Chi Exercise on Immunity and Infections: A Systematic Review of Controlled Trials. J Altern Complem Med. 2013;19(5):389–96 8p. 10.1089/acm.2011.0593 23317394

[63] Zheng G, Li S, Huang M, Liu F, Tao J, Chen L (2015). The effect of Tai Chi training on cardiorespiratory fitness in healthy adults: a systematic review and meta-analysis. Plos One.

[64] Hawkes TD, Manselle W, Woollacott MH (2014). Cross-sectional comparison of executive attention function in normally aging long-term T'ai chi, meditation, and aerobic fitness practitioners versus sedentary adults. J Altern Complem Med..

[65] Yang G (2012). Tai chi Chuan for youth. J Youth Sport.

[66] Sallis JF, Owen N, Glanz K, Rimer BK, Viswanath K (2015). Ecological models of health behaviour. Health behavior; theory, research, and practice.

[67] Atkinson NL, Permuth-Levine R (2009). Benefits, barriers, and cues to action of yoga practice: a focus group Approach. Am J Health Behav.

[68] Birdee GS, Legedza AT, Saper RB, Bertisch SM, Eisenberg DM, Phillips RS (2008). Characteristics of yoga users: results of a national survey. J Gen Intern Med.

[69] Jette M, Sidney K, Blumchen G (1990). Metabolic equivalents (METS) in exercise testing, exercise prescription, and evaluation of functional capacity. Clin Cardiol.

